# Reticulated Platelet Count as a Diagnostic Tool in Immune Thrombocytopenia (ITP)

**DOI:** 10.7759/cureus.41346

**Published:** 2023-07-04

**Authors:** Aqsa Javed Butt, Uzma Zaidi, Rabeea Munawar Ali, Sidra Zafar, Muhammad Shujat Ali, Tahir Shamsi

**Affiliations:** 1 Clinical Hematology, National Institute of Blood Diseases and Bone Marrow Transplantation, Karachi, PAK; 2 Research and Development, National Institute of Blood Diseases and Bone marrow Transplantation, Karachi, PAK

**Keywords:** thrombocytopenia, platelet counts, immune thrombocytopenia, immature platelet fraction, bone marrow

## Abstract

Objective: This study aimed to compare the reticulated platelet count between patients having thrombocytopenia secondary to autoimmune destruction (immune thrombocytopenia {ITP}), bone marrow failure, and healthy controls who presented to a tertiary care hospital in Karachi, Pakistan.

Methodology: A cross-sectional study was conducted from February 2021 to October 2022 in the Department of Hematology, National Institute of Blood Disease (NIBD) Hospital in Karachi, Pakistan, that involved examining three groups: 30 patients with immune thrombocytopenia, 30 patients with thrombocytopenia secondary to reduced production from bone marrow, and 30 healthy controls. The study utilized the Sysmex XN-1000 (Hyogo, Japan: Sysmex Corporation) automated hematology analyzer to perform a complete blood count (CBC) test. Additionally, peripheral blood was stained with Leishman stain and examined under a microscope to eliminate pseudo thrombocytopenia and identify any abnormal cells or dysplasia. The immature platelet fraction (IPF) was then performed on Sysmex XN 1000 after ensuring adequate quality control. Finally, the data were analyzed using DATAtab (Graz, Austria: DATAtab) and SPSS version 25 (Armonk, NY: IBM Corp.).

Results: Of the ninety participants, the median age was 33 years with a range of 18-71 years. Patients with ITP had a significantly higher median IPF% (median=26.65, IQR=15-39.4) than thrombocytopenia due to bone marrow failure (median=9.25, IQR=4.55-14.30) and healthy controls (median=7, IQR=4.40-9.90), with a p-value of 0.001. The immune thrombocytopenia group demonstrated an increase in IPF% as platelet counts increased, indicating a significant moderate correlation between IPF% and platelets in these patients (r=0.438, p=0.016) and confirming that IPF% was an independent predictor for the detection of ITP.

Conclusion: Reticulated platelet count may be a useful diagnostic tool to differentiate between ITP and thrombocytopenia caused by bone marrow failure. Because of its non-invasive nature, IPF is a valuable tool for expediting the management of thrombocytopenia associated with increased IPF.

## Introduction

Thrombocytopenia is a clinical condition characterized by a reduction in number of mature platelets in circulation. It is a common condition that can be caused by bone marrow failure, medication, and autoimmune disorders [1,2]. One such autoimmune disorder is immune thrombocytopenia (ITP), where autoantibodies bind to specific membrane glycoproteins causing premature destruction of platelets. Both children and adults can be affected by ITP, which is marked by a persistent or transient decrease in platelet numbers, resulting in an increased risk of bleeding [3]. Moreover, the global incidence rate of acute ITP ranges from 1.9 to 6.4 per 100,000 children and 3.3 per 100,000 adults annually [2].

Reticulated platelets or immature platelet fraction (IPF), is an automated parameter that measures the immature platelets as a fraction of the total number of platelets in the peripheral blood [4]. Reticulated platelets or IPF%, with their RNA content and larger size, can be differentiated from mature platelets and are thought to mimic the properties of red cell reticulocytes [5]. These platelets reflect the bone marrow's ability to generate platelets and may be used as a surrogate marker for megakaryocytic activity [5]. According to studies, IPF is raised in patients with peripheral platelet destruction and decreased or normal in people who have central thrombocytopenia [2,6,7].

Traditional diagnostic methods to evaluate the cause of thrombocytopenia, such as bone marrow biopsy, can be invasive, painful, and time-consuming [4]. This highlights the need for a non-invasive, low-cost diagnostic method like IPF to improve the accuracy and efficiency of diagnosis and facilitate better management of patients with ITP [1,2]. In this study, we aimed to compare the IPF values between patients with thrombocytopenia secondary to bone marrow failure, ITP, and controls. By establishing a reliable and non-invasive diagnostic method, invasive procedures can be avoided, improving patient care and reducing costs.

## Materials and methods

This cross-sectional research was carried out at the Department of Hematology of the National Institute of Blood Disease and Bone Marrow Transplant (NIBD), Karachi, Pakistan, from February 2021 to October 2022 after permission from the institutional review board (IRB #225/27-2021). A sample size of 83≈90 patients was estimated using the OpenEpi sample size calculator. Statistics considered for estimation were mean IPF value of 16.4% of ITP patients [2], margin of error of 8/%, and 95% confidence level. All patients aged more than 18 years of either gender were included in the study. The study included three groups - patients diagnosed with ITP (n=30), patients with thrombocytopenia secondary to bone marrow failure (n=30), and healthy controls (n=30). All patients were diagnosed based on history clinical examination, bone marrow biopsy, and other relevant tests. Patients with a history of liver or kidney disease, myeloproliferative disorders, megaloblastic anemia, lymphoproliferative disorders, bone marrow failure secondary to chemotherapy, thrombotic microangiopathies, pseudo thrombocytopenia, a recent history of blood transfusions, and patients taking medications known to affect platelet counts were excluded from the study. Samples were included in the study using a consecutive sampling technique.

Written informed consent was taken. Each participant's demographic information, including age and sex, was gathered. Each participant provided a blood sample of 5 mL, which was collected aseptically and stored in an ethylenediaminetetraacetic acid (EDTA) vial. The study utilized the Sysmex XN-1000 (Hyogo, Japan: Sysmex Corporation) automated hematology analyzer to perform a complete blood count (CBC) test. Additionally, peripheral blood was stained with Leishman stain and examined under a microscope to eliminate pseudo thrombocytopenia and identify any abnormal cells or dysplasia [1,8]. The reticulated platelet count (IPF%) was then performed on Sysmex XN 1000 after ensuring adequate quality control.

Data were analyzed using SPSS (Armonk, NY: IBM Corp.) version 25 and online DATAtab (Graz, Austria: DATAtab). The normality of the numeric data was assessed using Shapiro-Wilk’s test. Non-parametric numeric data like age, hemoglobin, total leukocyte count (TLC), platelets, and reticulated platelet count were presented as median and interquartile range (IQR). Categorical data like gender were presented as frequency and percentage. Comparison of numeric data between three groups was done using Kruskal-Wallis test and post-hoc pair-wise comparison was done. Comparison of categorical data between three groups was done using Pearson’s chi-square test. A p-value≤0.05 was considered significant.

## Results

The median age of the 90 participants was 33 years, with a range of 18-71 years. Fifty-seven (63.3%) patients were women, while 33 (36.7%) were men. The median hemoglobin level was 10.95 g/dL, TLC was 8.6×10^9^/L, and platelet count was 61.5×10^9^/L. The median platelet count was remarkably different among the three groups (p=0.001). Thrombocytopenia secondary to bone marrow failure and controls (p=0.010), thrombocytopenia secondary to bone marrow failure and ITP (p=0.016), as well as ITP and controls (p=0.010) all had significantly different median platelet counts (Table 1).

**Table 1:** Descriptive analysis of study participants (n=90). *P-value 0.05 was considered significant. TLC: total leukocyte count Data were presented as median (IQR) or n (%).

Characteristics		Overall	Immune thrombocytopenia	Bone marrow failure	Controls	p-Value
Age (years)		33 (18-71)	38.5 (22-50)	30 (21-50)	30 (23-47)	0.288
Gender	Male	33 (36.7)	10 (33.3)	13 (43.3)	10 (33.3)	0.650
	Female	57 (63.3)	20 (66.7)	17 (56.7)	20 (66.7)	
Hemoglobin (g/dL)		10.95 (7.65-12.32)	12 (10.5-12.9)	6.5 (5.3-8.9)	11.6 (10.8-12.5)	0.001*
TLC ×10^9^		8.6 (5.6-12.7)	9.79 (7.2-14)	5.7 (4.24-13.43)	8.05 (6.5-9.91)	0.079
Platelets ×10^9^/L		61.5 (15-190.25)	40.5 (10-88)	15 (6-36)	237 (189-332)	0.001*

**Figure 1:**
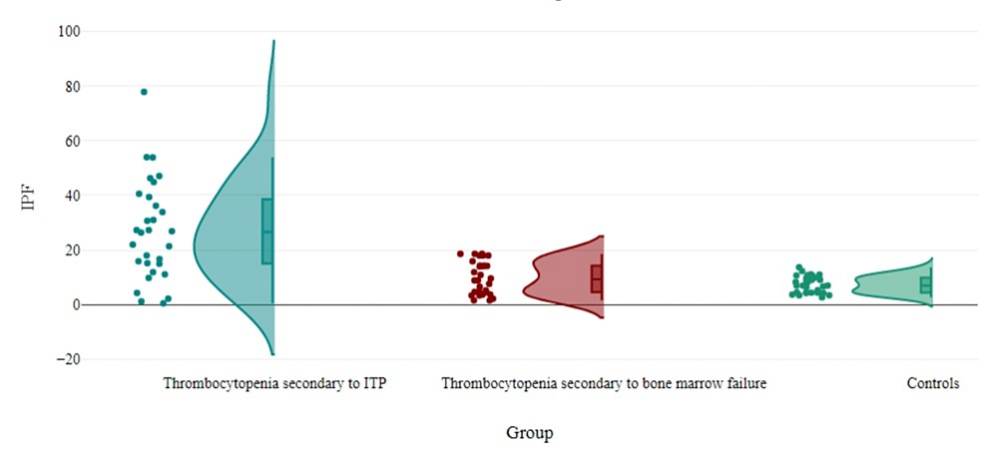
Comparison of IPF% in three groups. IPF: immature platelet fraction; ITP: immune thrombocytopenia

Figure 1 shows that with a p-value of 0.001, patients with thrombocytopenia secondary to ITP had a substantially greater median IPF% among groups. Sub-group analysis in Table 2 shows that the median IPF% was highest in thrombocytopenia secondary to ITP (median=26.65, IQR=15-39.4), followed by acute lymphoblastic leukemia (ALL) (median=14.10, IQR=6.57-18), acute myeloid leukemia (AML) (median=8.9, IQR=4.13-6.15), aplastic anemia (AA) (median=7.65, IQR=4.7-10.89), and controls (median=7, IQR=4.4-9.9). A pair-wise comparison revealed a significant difference in the median IPF% between thrombocytopenia secondary to ITP and controls (p=0.001) and aplastic anemia and thrombocytopenia secondary to ITP (p=0.009).

**Table 2:** Comparison of IPF% among sub-groups (n=90). *P-value 0.05 was considered significant. ATP: immune thrombocytopenia; ALL: acute lymphoblastic leukemia; AML: acute myeloid leukemia; AA: aplastic anemia; IPF: immature platelet fraction; ITP: immune thrombocytopenia

Groups		IPF%	p-Value
ITP (n=30)		26.65 (15-39.4)	0.001*
Thrombocytopenia secondary to decrease bone marrow production	ALL (n=10)	14.1 (6.57-18)	
	AML (n=11)	8.9 (4.12-16.15)	
	AA (n=9)	7.65 (4.7-10.89)	
	Controls (n=30)	7 (4.4-9.9)	

**Figure 2:**
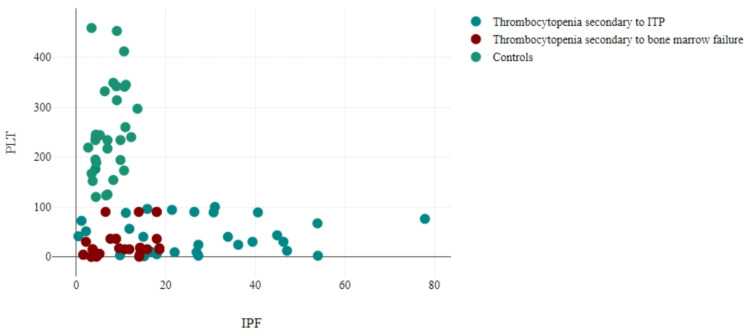
Correlation between platelet counts and IPF% among three groups (n=90). IPF: immature platelet fraction; ITP: immune thrombocytopenia; PLT: platelet count

The ITP group showed a decrease in IPF% with an increase in platelet counts; however, no significant correlation between IPF% and platelets was observed in these patients (r = -0.101, p = 0.597). On the other hand, the thrombocytopenia secondary to bone marrow failure group demonstrated an increase in IPF% as platelet counts increased, indicating a significant moderate correlation between IPF% and platelets in these patients (r=0.438, p=0.016). In the control group, IPF% also increased with an increase in platelet counts, but the correlation between IPF% and platelets was insignificantly moderate (r=0.347, p=0.060) (Figure 2).

## Discussion

In the past, bone marrow biopsy was used to confirm ITP; however, this technique is painful, and the tests can be time-consuming [9,10]. Later, the diagnosis was confirmed using flow cytometric tests, but they also have limitations [11]. Nowadays, automated quantification of reticulated platelets is performed in order to differentiate between ITP and other causes of thrombocytopenia [9,12]. This parameter can help with patient response monitoring, particularly when immunosuppressive medicine is changed [2]. Information on bone marrow megakaryocytic activity and platelet production may be obtained quickly and easily using IPF determination. Also, it can serve as a marker for platelet recovery in people who have received hematopoietic stem cell transplants or chemotherapy. Studies on these functions have been published [2,3,9,13]. Thus, we conducted a study on 90 participants to compare IPF% among three groups, i.e., thrombocytopenia secondary to autoimmune destruction (ITP), bone marrow failure, and healthy controls.

In the current research, when compared to patients with thrombocytopenia related to bone marrow failure and healthy controls, patients with thrombocytopenia secondary to autoimmune destruction had considerably greater median IPF% (p=0.001). In a related investigation, Naz et al. discovered that patients with ITP had considerably higher mean IPF% values than those without ITP [2]. According to Jeon et al., patients with central thrombocytopenia had considerably lower IPF levels than those with destructive/consumptive thrombocytopenia [14]. In another study by Ali et al., IPF% was shown to be considerably greater in cases of excessive platelet consumption or pseudo thrombocytopenia compared to healthy controls [15]. A study by Michelson demonstrated elevated IPF% in ITP patients than in bone marrow failure [16]. Ashraf et al. in their study found that patients with central thrombocytopenia had a median IPF of 8.2 (IQR=4.6-16.7), which was substantially lower than patients with peripheral thrombocytopenia, who had a median IPF of 25.5 (IQR=15.2-39.3) with a p-value of 0.001. IPF can thus be used to distinguish between central and peripheral thrombocytopenia, according to this study [7]. Additionally, Li et al. discovered that the IPF% was significantly lower in the group with reduced platelet production compared to the control group. They concluded that IPF may be a useful marker for identifying platelet destruction as a cause of thrombocytopenia [17]. Thus, taken together, the findings of these studies support the use of IPF% as a valuable tool for diagnosing ITP and other thrombocytopenic conditions, as well as differentiating between different types of thrombocytopenia.

The present study discovered a correlation between IPF% and platelet counts in thrombocytopenia secondary to autoimmune destruction. Bhat and Pai obtained comparable findings, noting that IPF was the most significant platelet parameter in thrombocytopenic patients and that a negative correlation between platelet counts and IPF% was observed in patients with ITP and thrombocytopenia but not in healthy individuals [18]. In contrast, Mampilly et al. in their study reported a moderate inverse correlation between platelets and IPF% in all etiologies of thrombocytopenia but did not provide specific findings for ITP, thrombocytopenia, and healthy controls separately [19]. Pons et al. identified a high association between IPF in patients with thrombocytopenia caused by peripheral platelet destruction but not in individuals with central thrombocytopenia or peripheral non-ITP by improper distribution. A strong association between IPF and reticulated platelets was also seen in the control group with normal platelets. These results generally indicated that IPF might be a useful screening method in individuals with isolated thrombocytopenia to separate peripheral platelet loss from other thrombocytopenia causes [20]. Clinicians should consider the individual patient's medical history and clinical presentation when interpreting these results [6,21].

Our study has several strengths and limitations. The study focuses on thrombocytopenia and improve patient management by providing non-invasive and efficient diagnostic tool. The study design is cross-sectional that uses a reliable and valid diagnostic tool, IPF, to assess the number of immature platelets in peripheral blood samples. The study has a small sample size of 90 participants and was conducted in a single center, which may limit the generalizability and external validity of the findings. The study did not evaluate the diagnostic accuracy of IPF in distinguishing between thrombocytopenia secondary to bone marrow failure and ITP, nor investigate the effect of medications on platelet counts or the relationship between IPF values and bleeding risk in patients with thrombocytopenia. The study findings can be utilized to develop new diagnostic strategies for ITP, and further research can be conducted to validate the use of IPF as a diagnostic tool for ITP in larger and more diverse patient populations. Overall, the study has important implications for improving the management and outcomes of patients with ITP.

## Conclusions

Immature platelet fraction may be a useful diagnostic tool for differentiating between thrombocytopenia secondary to bone marrow failure and ITP. The non-invasive nature of IPF makes it a valuable tool for improving patient management and facilitating timely diagnosis of ITP.
